# Mechanisms of Antibacterial Action of Quinoxaline 1,4-di-*N*-oxides against *Clostridium perfringens* and *Brachyspira hyodysenteriae*

**DOI:** 10.3389/fmicb.2016.01948

**Published:** 2016-12-05

**Authors:** Fanfan Xu, Guyue Cheng, Haihong Hao, Yulian Wang, Xu Wang, Dongmei Chen, Dapeng Peng, Zhenli Liu, Zonghui Yuan, Menghong Dai

**Affiliations:** ^1^National Reference Laboratory of Veterinary Drug Residues (HZAU), Ministry of Agriculture Key Laboratory for the Detection of Veterinary Drug Residues in Foods, Huazhong Agricultural UniversityWuhan, China; ^2^Ministry of Agriculture Laboratory for Risk Assessment of Quality and Safety of Livestock and Poultry Products, Huazhong Agricultural UniversityWuhan, China

**Keywords:** quinoxaline 1, 4-di-*N*-oxides, *Clostridium perfringens*, *Brachyspira hyodysenteriae*, cell wall, cell membrane, DNA damage

## Abstract

Quinoxaline 1,4-di-*N*-oxides (QdNOs) are a class of bioreductive compounds, however, their antibacterial mechanisms are still unclarified. The aim of this study was to assess the ability of two representative QdNO drugs, cyadox (CYA) and olaquindox (OLA), to produce reactive oxide species (ROS) in Gram-positive anaerobe *Clostridium perfringens* CVCC1125 and Gram-negative anaerobe *Brachyspira hyodysenteriae* B204. In addition, the effects of QdNOs on the integrity of bacterial cell walls and membranes as well as the morphological alterations and DNA oxidative damage in *C. perfringens* and *B. hyodysenteriae* were analyzed. It was demonstrated that under anaerobic conditions, QdNOs were metabolized into the reduced products which did not show any antibacterial activity. A significant dose-related increase of intracellular ROS level and intracellular hydroxyl radicals were evident in bacteria exposed to QdNOs. The result of biochemical assay showed that the cell walls and membranes of the bacteria treated with QdNOs were damaged. After exposure to 1/2MIC to 4MIC of CYA and OLA, *C. perfringens* and *B. hyodysenteriae* became elongated and filamentous. Morphological observation with scanning and transmission electron microscopes revealed rupture, loss of cytoplasmic material and cell lysis in QdNO-treated bacteria, indicating serious damage of cells. There was an increase of 8-OHdG in the two strains treated by QdNOs, but it was lower in *C. perfringens* CVCC1125 than in *B. hyodysenteriae* B204. Agarose gel electrophoresis showed the degradation of chromosomal DNA in both of the two anaerobes treated by QdNOs. The results suggest that QdNOs may kill *C. perfringens* and *B. hyodysenteriae* via the generation of ROS and hydroxyl radicals from the bacterial metabolism of QdNOs, which cause oxidative damage in bacteria under anaerobic conditions.

## Introduction

Quinoxaline 1,4-di-*N*-oxide derivatives (QdNOs), including carbadox, OLA, mequindox, quinocetone, and CYA, are a class of synthetic heterocycles that are known as potent antibacterial agents against many Gram-positive and Gram-negative bacteria, especially anaerobes. Carbadox, OLA, mequindox are commonly used as feed additives to prevent bacterial infectious disease and improve animal growth in livestock and poultry (Cheng et al., 2016).

*Clostridium perfringens* is a Gram-positive spore-forming obligate anaerobe that causes food poisoning, gas gangrene, and antibiotic-associated diarrhea (Farzan et al., 2013; Uzal et al., 2014). Swine dysentery (SD) is a common disease caused by the anaerobic intestinal *Brachyspira hyodysenteriae* among pigs worldwide (Moxley and Duhamel, 1999), which contributes to major production losses. Recently, an increase of antimicrobial resistance to antibiotics used for routine treatment of SD has been observed in most European countries (Alvarez-Ordonez et al., 2013), and this may be caused by excessive use of antibiotics (Domadia et al., 2008). *C. perfringens* with decreased susceptibility to metronidazole (Alvarez-Perez et al., 2016) was found to be sensitive to CYA and OLA in the study of our laboratory. Besides, carbadox used to be added to feed or drinking water for the treatment of SD (Sabbatini, 2009).

However, carbadox, OLA, and mequindox were shown to have genetic and carcinogenic toxicity to eukaryotic cell (Liu et al., 2016). CYA is a novel QdNO derivative, and is a safe member of the QdNO family according to the microbiological safety evaluation of CYA (Hao et al., 2013).

Previous studies have revealed that QdNOs are DNA synthesis inhibitors, which can cause cytotoxic DNA strand breaks in *Escherichia coli* (Suter et al., 1978; Ganley et al., 2001; Cheng et al., 2015). There is no evidence that QdNOs can covalently bind to DNA (Ganley et al., 2001). Though recent study has provided support for the involvement of free radicals induced by QdNOs in *E. coli* (Cheng et al., 2015), it remains unclear how QdNOs modify the structure of DNA. Moreover, the existing knowledge about the antibacterial action of QdNOs has only been studied in *E. coli*, and little is known about their antibacterial modes in Gram-positive bacteria and anaerobes.

In this study, the mechanisms of the antibacterial action of QdNOs against Gram-positive anaerobe *C. perfringens* and Gram-negative anaerobe *B. hyodysenteriae* were investigated. The measurement of free radicals in Gram-positive bacteria and anaerobes treated by QdNOs was complementary to the study of antibacterial mechanism of QdNOs in *E. coli*. The effects of QdNOs on the integrity of cell walls and cell membrane as well as DNA damage were analyzed. This study provides new insights into the effectiveness and the underlying mechanism of QdNOs against anaerobes to contribute to the development of new QdNOs.

## Materials and Methods

### Drugs and Chemicals

Cyadox, bisdesoxycyadox (Cy1), Cy2 and Cy10, OLA, olaquindox-4-monoxide (O1), olaquindox-1-monoxide (O7), and bisdesoxyolaquindox (O2) (**Table 1**) were synthesized by the Institute of Veterinary Pharmaceuticals (HZAU) (Wuhan, China). ALP detection kit and ROS assay kit were obtained from Beyotime (Shenzhen, China). 3′-(p-hydroxyphenyl) fluorescein (HPF) was acquired bought from Invitrogen (Carlsbad, CA, USA). DNeasy Blood and Tissue Kit was obtained from QIAGEN (Nasdaq, Germany). OxiSelect Oxidative DNA Damage ELISA Kit was obtained from Cell Biolabs (Santiago, USA). All other chemicals and reagents commercially available were of the highest analytical grade.

**Table 1:** The structures of QdNOs and their metabolites.

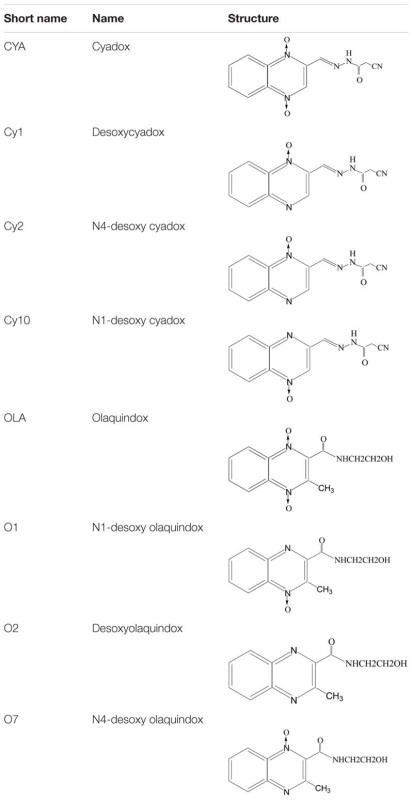

### Bacteria Strains and Culture Conditions

Pig-origin *C. perfringens* CVCC1125 (type A) was obtained from the China Institute of Veterinary Drug Control (IVDC) and *B. hyodysenteriae* B204 (ATCC31212) was acquired from the ATCC.

*Clostridium perfringens* CVCC1125 and *B. hyodysenteriae* B204 cells were incubated, respectively, on trypticase soy agar (TSA) plates supplemented with 5% sheep blood and BHI broth supplemented with 10% fetal bovine serum broth anaerobically (80% N_2_, 10% H_2_, 10% CO_2_) at 37°C for 24 or 72 h as previously described before (Lugsomya et al., 2012).

### Antibacterial Susceptibility Test

The MICs of QdNOs and their metabolites against *C. perfringens* CVCC1125 and *B. hyodysenteriae* B204 were determined by the agar dilution method according to the M11-A7 Clinical and Laboratory Standards Institute (CLSI) (CLSI, 2007).

### Intracellular ROS and Hydroxyl Radical Measurement

The bacterial culture (about 10^7^ CFU/mL, McFarland standard 0.5 for *C. perfringens and* McFarland standard 1 for *B. hyodysenteriae*) was treated with QdNOs at different concentrations under anaerobic conditions and incubated for indicated times at 37°C. The reactions were stopped at 4°C. DCFH-DA was used to detect intracellular ROS in *C. perfringens* and *B. hyodysenteriae*, respectively, by fluorescence microplate reader according to the a prior study (Cheng et al., 2015). The samples were analyzed for ROS generation using fluorescence excitation and emission wavelengths at 488 and 525 nm by fluorescence microplate reader, respectively.

3-(*p-*aminophenyl) fluorescein (APF) (Invitrogen, Canada) was used for the detection of hydroxyl radicals in bacteria treated by QdNOs. A generation of hydroxyl radical was monitored by adding 10 μM of APF to each tube containing bacterial inoculum and drug dilutions (5 mL). The samples were analyzed for hydroxyl radical generation using fluorescence excitation and emission wavelengths at 490 and 530 nm by fluorescence microplate reader, respectively.

2.5 mM (85 μg/mL) H_2_O_2_ was used as positive control for *C. perfringens* CVCC1125 and 300 μM (10.2 μg/mL) H_2_O_2_ was used as positive control for *B. hyodysenteriae* B204 (Stanton et al., 2008b).

### Cell Wall Integrity Measurement

Alkaline phosphatase is an enzyme present in the periplasmic space of the bacteria (Arokiyaraj et al., 2015). ALP was measured in QdNO-treated bacterial culture (about 10^6^ CFU/mL) using an ALP assay kit (Beyotime, Shenzhen, China). This assay involved a colorimetric determination of para-nitrophenol (p-nitrophenol) released from para-nitrophenyl phosphate (pNPP) with absorption which can be detected at 405 nm by microplate reader. After incubation, cell free supernatants were collected. All the treatments were compared with control wells (cells without treatment) and the final results were expressed in units/liter (Arokiyaraj et al., 2015).

### Cell Membrane Integrity Measurement

Bacterial cell membrane integrity was examined by determining the release of intracellular materials as previously described (Eom et al., 2014). Bacterial cells (about 10^6^ CFU/mL) of *C. perfringens* and *B. hyodysenteriae* were harvested by centrifugation at 3500 rpm for 10 min and washed twice by 0.9% NaCl solution. The bacteria suspensions were exposed to QdNOs at the indicated concentrations and tested at 30 min intervals during 120 min. Subsequently the release of intracellular materials absorbing at 260 nm was determined using a UV spectrometer, which were interpreted to be mostly DNA and RNA (Chen and Cooper, 2002).

### Scanning Electron Microscopy (SEM)

Bacteria (about 10^6^ CFU/mL) were exposed to different concentrations of CYA or OLA at indicated time under anaerobic conditions. Enrofloxacin, a DNA-damaging fluoroquinolone which can induce SOS response leading to the filamentation of bacteria (Piddock et al., 1990), was used as a positive control drug. Samples were washed with PBS and fixed in 2.5% glutaraldehyde overnight at 4°C. After washing with PBS three times, the cells were dehydrated in a gradually increased concentration series (30, 50, 70, 85, and 95%) of ethanol solution for 15 min. The cells were then washed in 100% ethanol for 20 min twice before being freeze-dried and coated with gold and subjected to SEM analysis (JSM-6390LV, Japan) (Kumar Tyagi et al., 2013; Cheng et al., 2015).

### Transmission Electron Microscopy (TEM)

Bacteria (about 10^6^ CFU/mL) were exposed to QdNOs (enrofloxacin was used as a positive control drug), then were harvested by centrifugation, washed thrice with 0.1M PBS (pH 7.4), and fixed in 0.2% glutaraldehyde in 0.1 M cacodylate buffer for 4 h at 4°C, and then fixed in the perfluorocarbon containing 1% osmium tetroxide for 1 h. After three rinses in pure perfluorocarbon, the samples were dehydrated, embedded, sectioned, stained, and imaged with a TEM (H-7650, Japan) as previously described (Kumar Tyagi et al., 2013).

### Detection of QdNO-Induced Chromosomal DNA Damage

Bacterial cultures (about 10^6^ CFU/mL) in early exponential growth phase in BHI broth were treated with different concentrations of drugs. Enrofloxacin (2 μg/ml) was used as the positive DNA-damaging agent for *C. perfringens*, and 200 μM, (6.8 μg/mL) H_2_O_2_ was use as positive agent for *B. hyodysenteriae* by agarose gel electrophoresis (Matson et al., 2005).

OxiSelect Oxidative DNA Damage ELISA Kit (Cell Biolabs, Santiago, CA, USA) were used to detect DNA oxidative damage (Matson et al., 2005). 2.5 mM (85 μg/mL) H_2_O_2_ was used as the positive for *C. perfringens*, and 200 μM (6.8 μg/mL) H_2_O_2_ was use as positive agent for *B. hyodysenteriae.*

The DNeasy Blood and Tissue Kit (Qiagen, Nasdaq, Germany) was used to extract chromosomal DNA of *C. perfringens*, using the Gram-positive bacterial protocol, while the Gram-negative bacterial protocol of DNeasy Blood and Tissue Kit was used to extract *B. hyodysenteriae* chromosomal DNA as previously described (La et al., 2014). DNA was resuspended in sterile water and stored at -20°C.

### Statistical Analysis

All statistical analyses were performed using GraphPad Prism software (GraphPad Software Inc., La Jolla, CA, USA) with all data represented as the mean ± standard deviation (SD) from at least three independent experiments. Data analyses were done using the *t*-test or one-way analysis of variance (ANOVA), and *p-*value less than 0.05 was considered statistically significant.

## Results

### The Antibacterial Activities of QdNOs and Their Metabolites against *C. perfringens* and *B. hyodysenteriae*

Cyadox was metabolized into Cy1 and Cy2 in *C. perfringens* CVCC1125 and *B. hyodysenteriae* B204 under anaerobic conditions (Supplementary Figure S1). OLA was metabolized into O1 and O2 in these two species under anaerobic conditions (Supplementary Figure S2), indicating that QdNO was mainly reduced at the two *N*-oxide groups on the quinoxaline ring. The MICs for QdNOs against *C. perfringens* CVCC1125 and *B. hyodysenteriae* B204 are presented in **Table 2**. The MIC values of both CYA and OLA on *C. perfringens* CVCC1125 were 1 μg/ml. CYA and OLA had a MIC value of 0.031 and 0.0625 μg/ml for *B. hyodysenteriae* B204, respectively. Although both species were susceptible to QdNOs, the *N*-deoxy metabolites of QdNOs showed no antibacterial activity (MIC > 128 μg/ml) (**Table 2**), indicating that the two *N*-oxide groups were necessary for the antibacterial activity of QdNOs against these two anaerobes.

**Table 2:** Minimum inhibitory concentration of CYA, OLA and their metabolites against *C. perfringens* and *B. hyodysenteriae* under anaerobic conditions.

Drugs	*C. perfringens* CVCC1125	*B. hyodysenteriae* B204
CYA	1	0.031
Cy1	≥128	≥128
Cy2	≥128	≥128
Cy10	≥128	>128
OLA	1	0.0625
O1	≥128	≥128
O2	≥128	≥128
O7	≥128	≥128

*(Unit: μg/mL).*

*Clostridium perfringens* CVCC1125 growths measured by OD at 600 nm (the culture OD) treated with low concentrations CYA and OLA (0.5 and 1 μg/ml) were less affected compared to the groups treated with high concentration CYA and OLA (2 and 4 μg/ml) (Supplementary Figures S3A,B). *C. perfringens* CVCC1125 growth treated by 2 μg/ml CYA and OLA was greatly inhibited (little increase in the culture) from 0 to 4 h. *C. perfringens* CVCC1125 growth treated by 4 μg/ml CYA and OLA was greatly inhibited for nearly 6 h, but the culture OD increased slowly after that. *B. hyodysenteriae* B204 growth treated with 0.031 μg/ml CYA (Supplementary Figure S3C) and 0.0625 μg/ml OLA (Supplementary Figure S3D) was inhibited (a slower increase in the culture OD from 0 to 12 h compared to the untreated *B. hyodysenteriae* cells). *B. hyodysenteriae* B204 growth treated with high concentration QdNOs (0.125 μg/ml CYA and 0.25 μg/ml OLA) was greatly affected (the culture OD declined from 2 to 12 h), suggesting that the cells were killed. While, *B. hyodysenteriae* B204 growth treated by 0.016 μg/ml CYA and 0.031 μg/ml OLA was less influenced compared to *B. hyodysenteriae* cells treated with high concentration QdNOs. So QdNOs are concentration-dependent antibacterials for *C. perfringens* CVCC1125 and *B. hyodysenteriae* B204.

### QdNO-Induced ROS and Hydroxyl Radical Production

**FIGURE 1:**
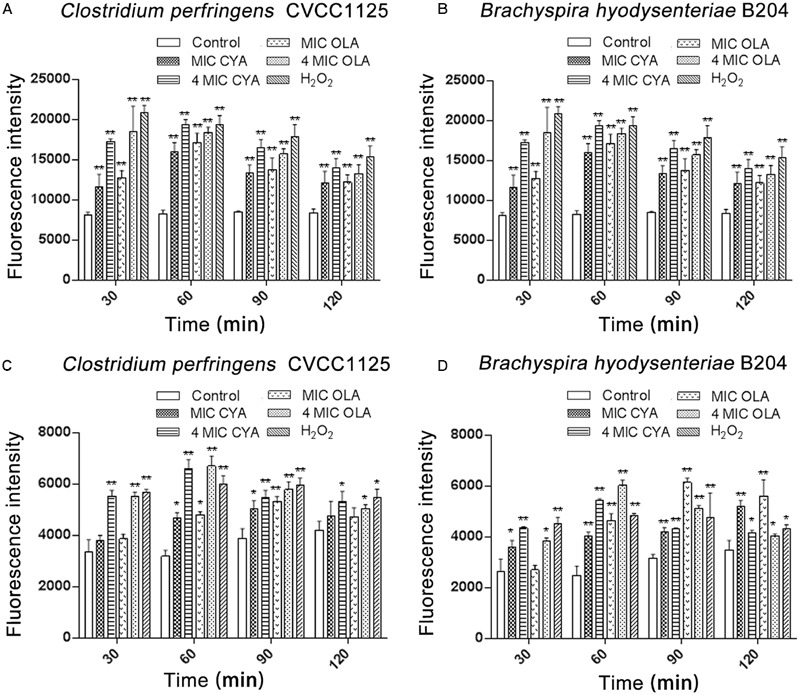
**Intracellular levels of ROS (A,B)** and hydroxyl radicals **(C,D)** in *Clostridium perfringens* and *Brachyspira hyodysenteriae.* **(A,C)** Under anaerobic conditions, *C. perfringens* CVCC1125 cells were treated with indicated concentration of CYA or OLA, and 2.5 mM (85 μg/mL) H_2_O_2_ was used as positive control. **(B,D)** Under anaerobic conditions, *B. hyodysenteriae* B204 cells were treated with indicated concentration of CYA or OLA, and 300 μM (10. 2 μg/mL) H_2_O_2_ was used as positive control. The levels of ROS **(A,B)** and hydroxyl radicals **(C,D)** were determined as described in the section “Materials and Methods.” Data were shown as mean ± SD (error bar), *n* = 3. ^∗^*p* < 0.05, ^∗∗^*p* < 0.01.

Under anaerobic conditions, ROS were detected in *C. perfringens* CVCC1125 and *B. hyodysenteriae* B204 exposed to QdNOs (**Figures 1A,B**). The levels of ROS in QdNOs or H_2_O_2_-treated cells were significantly higher than that of ROS in control cells from the time period of 30 to180 min.

Besides, under anaerobic conditions, QdNOs induced a release of hydroxyl radicals (**Figures 1C,D**), and the HPF fluorescence intensity in *C. perfringens* CVCC1125 was observed under fluorescence microscope (Supplementary Figure S4). The levels of hydroxyl radicals in QdNOs or H_2_O_2_-treated cells were significantly higher than the level of ROS in control cells.

The production of ROS and hydroxyl radicals increased in *C. perfringens* CVCC1125 or *B. hyodysenteriae* B204 incubated with an increasing concentration of QdNOs, with statistical significances between different doses, indicating that the ROS and hydroxyl radical formation were dose-dependent.

### Effect of QdNOs on the Integrity of Bacterial Cell Wall

**FIGURE 2:**
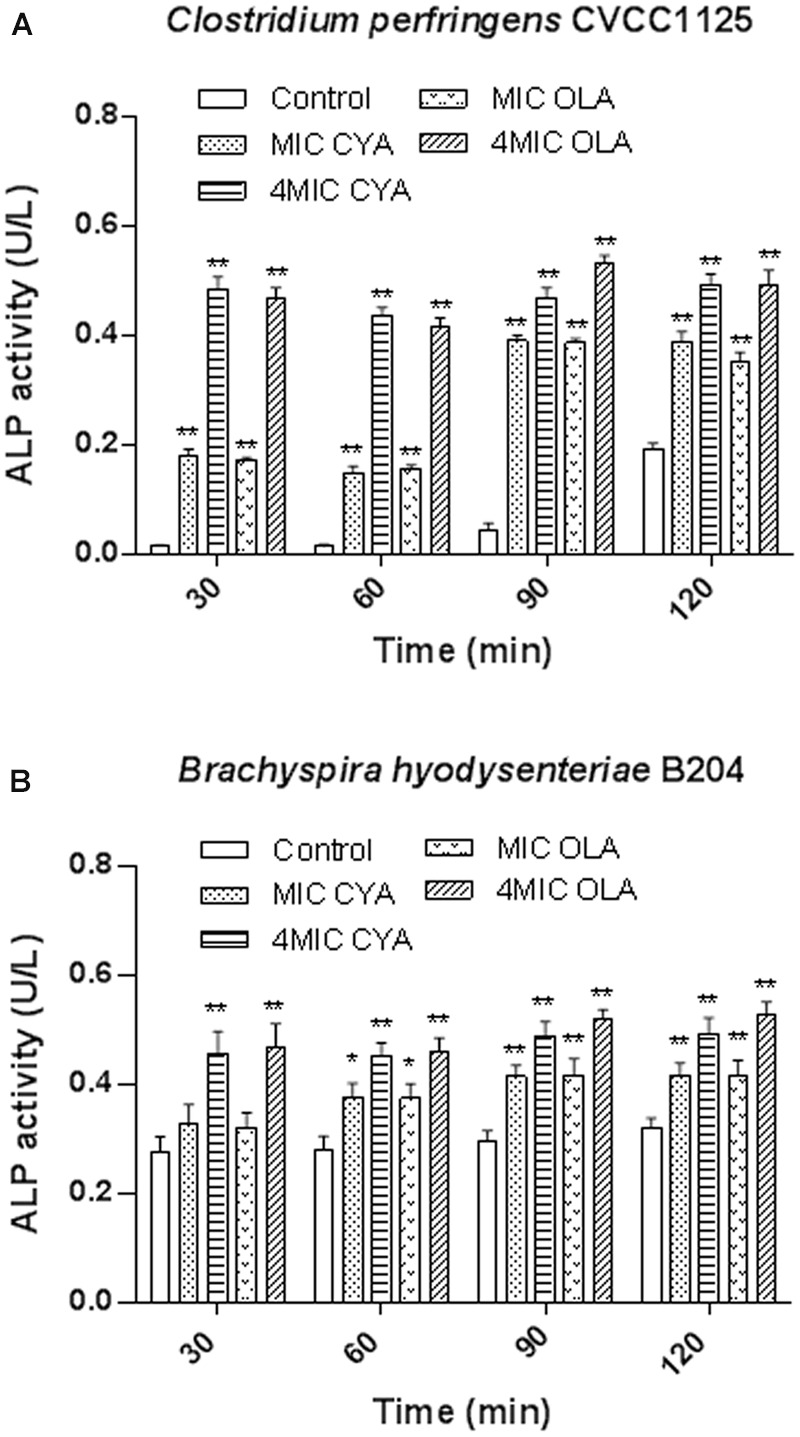
**The effects of QdNOs on the cell wall integrity of *C. perfringens* CVCC1125 (A)** and *B. hyodysenteriae* B204 **(B)**. **(A)** Under anaerobic conditions, *C. perfringens* CVCC1125 cells were treated with indicated concentration of CYA or OLA. **(B)** Under anaerobic conditions, *B. hyodysenteriae* B204 cells were treated with indicated concentration of CYA or OLA. The ALP level was determined by fluorescence microplate reader as described in the material and methods. Data were shown as mean ± SD (error bar), *n* = 3. ^∗^*p* < 0.05, ^∗∗^*p* < 0.01.

In our study, the ALP levels in the *C. perfringens* and *B. hyodysenteriae* cells were increased upon treatment with QdNOs compared to the non-treated groups (**Figure 2**). In the present results, the increase of the amount of ALP upon CYA and OLA addition was greater in *C. perfringens* than in *B. hyodysenteriae*, probably because that *C. perfringens* is a spore-forming obligate anaerobe, which produces higher amounts of ALP during phosphate starvation and sporulation (Tsai and Su, 1999; Sebastian and Ammerman, 2009).

### Effect of QdNOs on the Integrity of Bacterial Membrane

**FIGURE 3:**
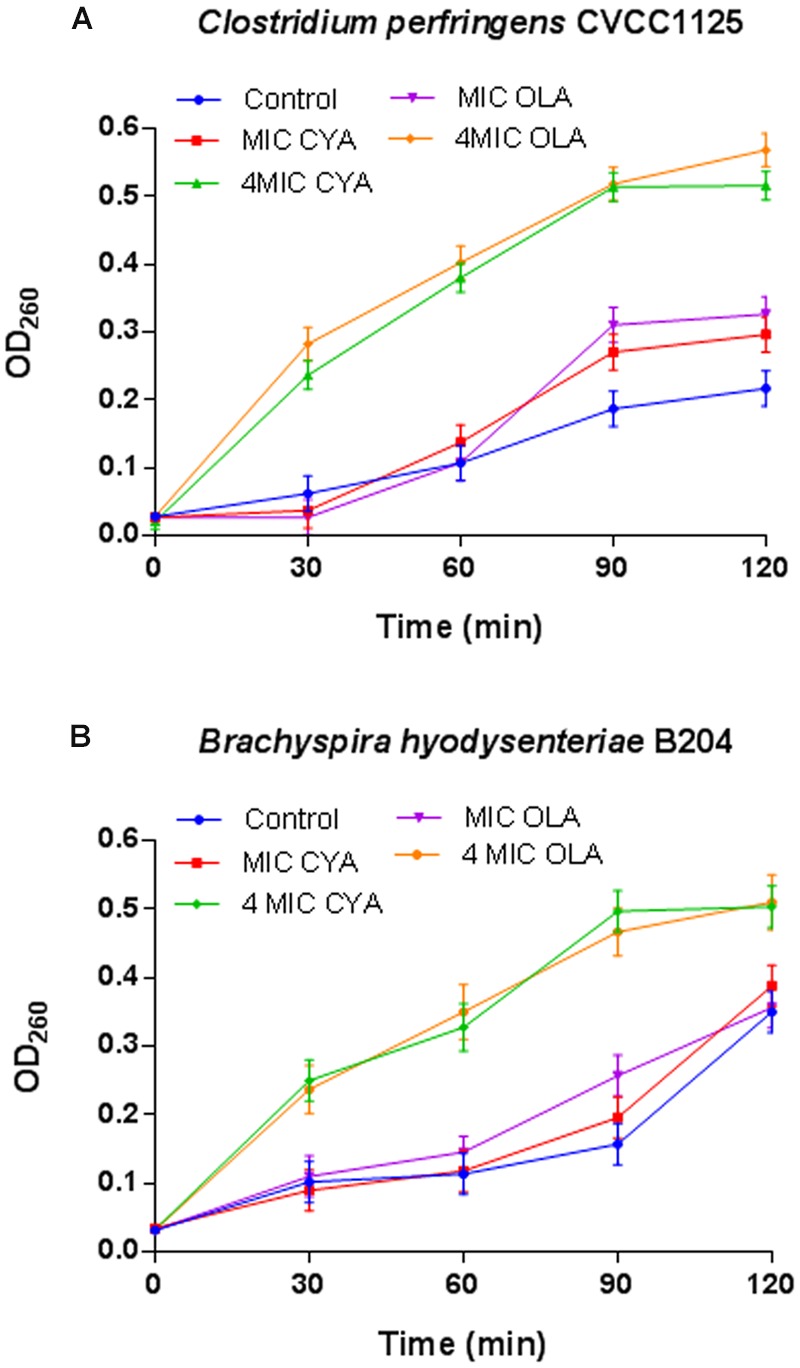
**Effects of QdNOs on cell membrane integrity of *C. perfringens* CVCC1125 (A)** and *B. hyodysenteriae* B204 **(B)**. **(A)** Under anaerobic conditions, *C. perfringens* CVCC1125 cells were treated with indicated concentration of CYA or OLA. **(B)** Under anaerobic conditions, *B. hyodysenteriae* B204 cells were treated with indicated concentration of CYA or OLA. The release of cell materials absorbing at 260 nm was determined by a UV spectrometer as described in the material and methods. Data were shown as mean ± SD (error bar), *n* = 3.

The release of cell materials absorbing at 260 nm, mainly DNA or RNA materials leaked from the cells. These results show that leakage of A260 absorbing material from the bacterial cells increased with increasing QdNOs concentration and exposure time (**Figure 3**). When *C. perfringens* cells and *B. hyodysenteriae* cells were treated with 4MIC CYA and 4MIC OLA, absorbance of the suspensions at 260 nm increased significantly up to120 min. But the leakage of A260 absorbing material from *C. perfringens* cells and *B. hyodysenteriae cells* treated with MIC CYA and MIC OLA increased slowly. So the release rate of the intracellular components showed a dose-dependent increasing tendency, suggesting that the membrane integrity was significantly affected by high concentration of QdNOs.

### Morphological Alterations of *C. perfringens* and *B. hyodysenteriae* Exposed to QdNOs

**FIGURE 4:**
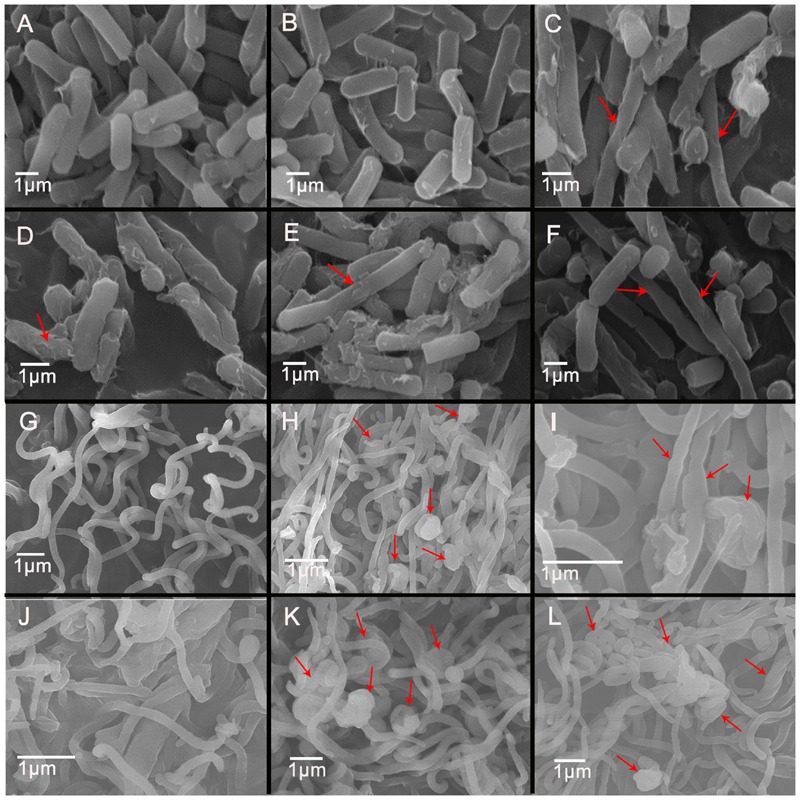
**Scanning electron microscopy of *C. perfringens* (A–F)** and *B. hyodysenteriae* **(G–L)** cells exposed to QdNOs. *C. perfringens* CVCC1125 cells were untreated **(A)** or treated with 0.08% DMSO **(B)**, 1 μg/mL CYA **(C)**, 4 μg/mL CYA **(D)**, 1 μg/mL OLA **(E)**, and 1 μg/mL enrofloxacin as positive control **(F)** under anaerobic conditions for 6 h. *B. hyodysenteriae* B204 cells were untreated **(G)** or treated with 0.031 μg/mL CYA **(H)**, 0.031 μg/mL CYA **(I)**, 0.125 μg/mL CYA **(J)**, and 0.0625 μg/mL OLA **(K)** and 8 μg/mL enrofloxacin as positive control **(L)** under anaerobic conditions for 6 h.

After exposure to 1/2MIC to 4MIC of CYA and OLA, *C. perfringens* CVCC1125 and *B. hyodysenteriae* B204 became elongated as observed by light microscope (Supplementary Figure S5) and SEM (**Figure 4**), indicating that the cell division was halted. *C. perfringens* CVCC1125 treated with CYA and OLA appeared longer, with rougher surface (**Figures 4C,F**), and became deformed and cracked (**Figures 4D,E**) in comparison to the control. The control appeared as intact rod shape and with a completely smooth surface (**Figure 4A**). The untreated *B. hyodysenteriae* B204 cells exhibited a typical smooth helical form with pointed ends which separated with each other (**Figure 4G**), while cells treated with CYA or OLA appeared to be aggregated, retracted (**Figures 4H,K,L**), partially deformed (**Figures 4I,J**), and with rough surface (**Figures 4H–L**).

**FIGURE 5:**
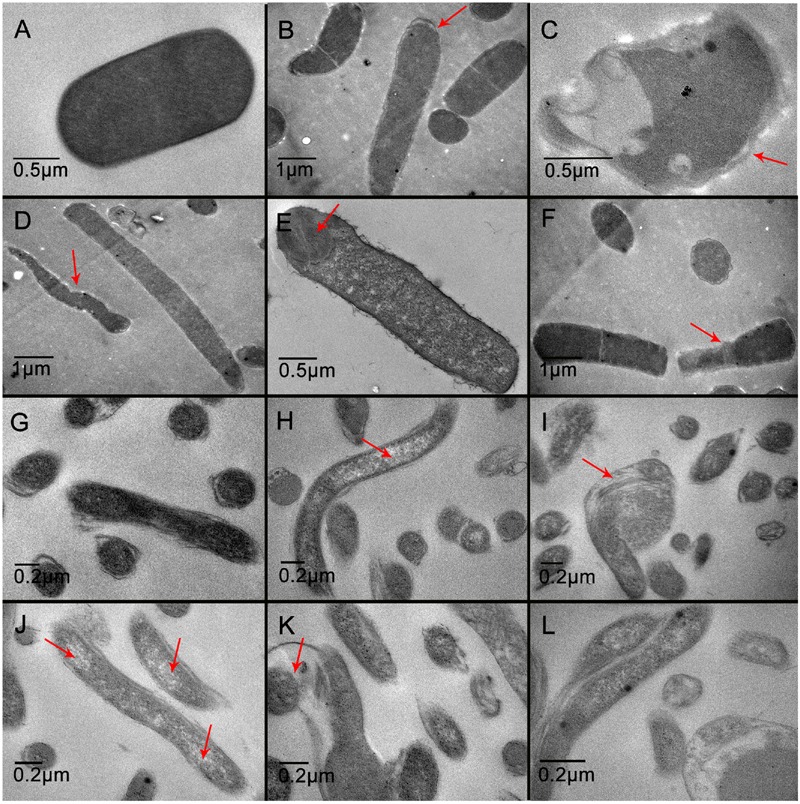
**Transmission electron microscopy of *C. perfringens* (A–F)** and *B. hyodysenteriae* **(G–L)** cells exposed to QdNOs. *C. perfringens* CVCC1125 cells were untreated **(A)** or treated with 1 μg/mL CYA **(B)**, 4 μg/mL CYA **(C)**, 1 μg/mL OLA **(D)**, 4 μg/mL OLA **(E)**, and 1 μg/mL enrofloxacin **(F)** as positive control under anaerobic conditions for 6 h. *B. hyodysenteriae* cells were untreated **(G)** or treated with 0.031 μg/mL CYA **(H)**, 0.125 μg/mL CYA **(I)**, 0.0625 μg/mL OLA **(J)**, 0.25 μg/mL OLA **(K)**, and 8 μg/mL enrofloxacin as positive control **(L)** under anaerobic conditions for 6 h.

Transmission electron microscopy demonstrated that the untreated *C. perfringens* cells showed a regular outlined cell wall, a cell membrane closely to the cell wall, regularly distributed cytoplasm (**Figure 5A**), and unusual cell division (**Figures 5B,F**) were observed. While some spores in QdNO-treated *C. perfringens* cells was also observed in almost all the treated groups, **Figure 5E** is shown for OLA-treated *C. perfringens*. Moreover, some *C. perfringens* cells showed wide range of abnormalities and extensive ultrastructure damages (**Figures 5C,D**). In *B. hyodysenteriae*, the untreated cells revealed regularly distributed cytoplasm and normal periplasmic space (**Figure 5G**), while QdNO-treated cells revealed a large loss of cytoplasmic material (**Figures 5H–L**) and irregular morphological structure (**Figures 5I,K**).

### QdNO-Induced DNA Damage in *C. perfringens* and *B. hyodysenteriae*

**FIGURE 6:**
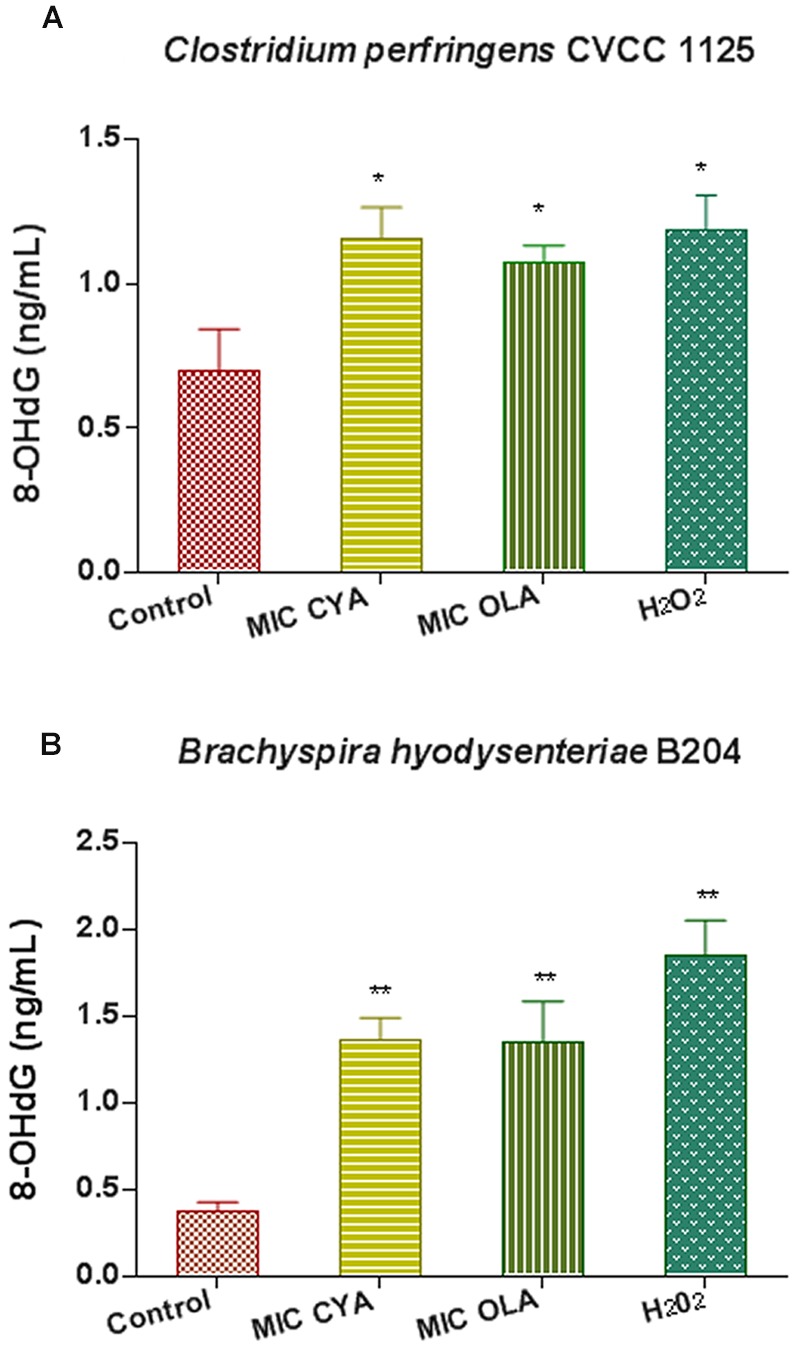
**Oxidative damage of DNA in *C. perfringens* (A)** and *B. hyodysenteriae* **(B)** exposed to QdNOs. **(A)** Under anaerobic conditions, *C. perfringens* CVCC1125 cells were treated with MIC of CYA and OLA, and 2.5 mM (85 μg/mL) H_2_O_2_ was used as positive control drug. **(B)** Under anaerobic conditions, *B. hyodysenteriae* B204 cells were treated with MIC of CYA and OLA, and 200 μM (6.8 μg/mL) H_2_O_2_ was used as positive control. The 8-OHdG level was detected by OxiSelect Oxidative DNA Damage ELISA Kit. Data were shown as mean ± SD (error bar), *n* = 3. ^∗^*p* < 0.05, ^∗∗^*p* < 0.01.

When *C. perfringens* CVCC1125 cells and *B. hyodysenteriae* B204 cells were incubated with QdNOs for 6 h, the 8-OHdG level significantly increased compared with the non-treated group (**Figure 6**). *B. hyodysenteriae* B204 cells produced higher level of 8-OHdG than that of *C. perfringens* CVCC1125 cells after treatment with QdNOs, probably because *B. hyodysenteriae* B204 was more sensitive to QdNOs, resulting in a low ability of resistance to oxidative damage.

**FIGURE 7:**
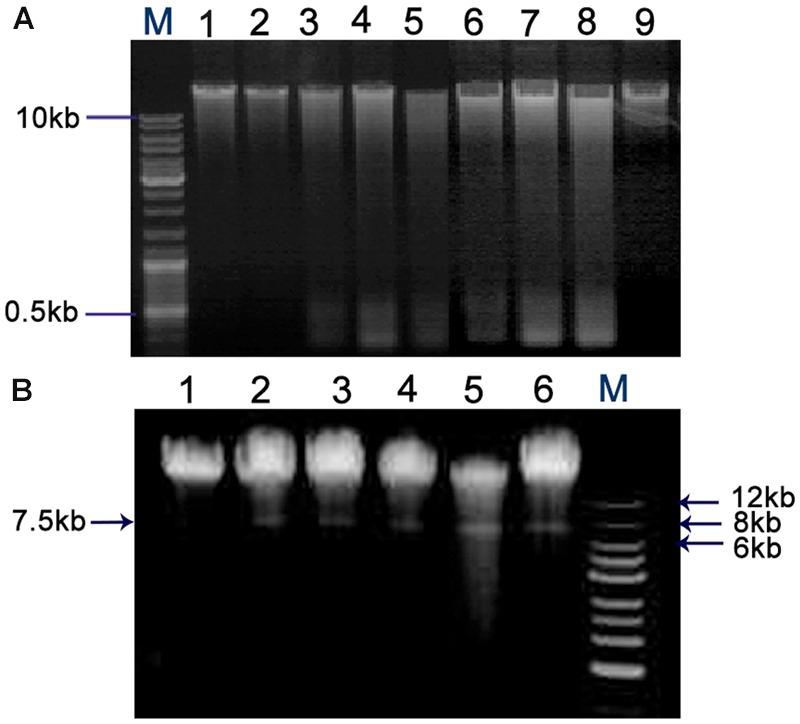
**Chromosome DNA damages in *C. perfringens* (A)** and *B. hyodysenteriae* cells **(B)** treated by QdNOs. **(A)** *C. perfringens* CVCC1125 cells incubated with different concentrations of CYA (lane 1, control; lane 2, 0.08% DMSO; lane 3, 1 μg/mL CYA; lane 4, 2 μg/mL CYA; lane 5, 4 μg/mL CYA); and OLA (lane 6, 1 μg/mL OLA; lane 7, 2 μg/mL OLA; lane 9, control) at 37°C for 6 h under anaerobic condition. 2 μg/mL enrofloxacin (lane 8) was used as the positive control drug. **(B)** *B. hyodysenteriae* B204 cells incubated with different concentrations of OLA CYA and (lane 1, control; lane 2, 0.0625 μg/mL OLA; lane 3, 0.031 μg/mL CYA; lane 4, 0.0625 μg/mL CYA; lane 5, 0.125 μg/mL CYA) at 37°C, for 6 h under anaerobic condition. 200 μM H_2_O_2_ (lane 6) was used as the positive control. Agarose gel electrophoresis was used to detect DNA damage. M, DNA maker.

The QdNO-induced DNA fragmentations are shown in **Figure 7**. The chromosome DNA showed laddering of DNA and the degradation degree increased with an increase of drug concentration in *C. perfringens* cells treated with QdNOs (**Figure 7A**). There was also a degradation of chromosome DNA in *B. hyodysenteriae* cells treated by QdNOs and H_2_O_2_ (**Figure 7B**). Besides, there was a band in the chromosome DNA of *B. hyodysenteriae* with a molecular size of about 7.5 kb, which is in accordance with the size of the purified bacteriophage designated VSH-1 (VSH for virus of *Serpulina hyodysenteriae*) (Humphrey et al., 1995; Stanton et al., 2008a,b).

## Discussion

Quinoxaline 1,4-di-*N*-oxides belong to the heterocyclic family of benzodiazepine with its N–O groups situated at 1- and 4-positions which are considered to contribute to antibacterial activities and other versatile abilities (Hennessey and Edwards, 1972; Cheng et al., 2016). In this study, we have presented evidence that CYA and OLA were reduced in *C. perfringens* and *B. hyodysenteriae* cells and their reduced products did not show any antibacterial activity under anaerobic conditions (**Table 2**), indicating that the reduction potential of QdNOs, which may generate free radical intermediates, contributes to their antibacterial action, consistent with the earlier report studied in *E. coli* (Cheng et al., 2015). Like tirapazamine (TPZ) (Shinde et al., 2010), enzymatic reduction of QdNOs under hypoxic conditions results in a one-electron reduction product that has been characterized as a free radical intermediate but its exact structure is not clear. The free radical intermediate is unstable, and in the presence of oxygen, the active intermediate is oxidized back to non-toxic prototypical drug (Silva and O’Brien, 1993). This is thought to be the basis for the selective toxicity of QdNOs under anaerobic conditions. Xanthine oxidase is one of the QdNO metabolic enzymes in bacteria (Ganley et al., 2001; Cheng et al., 2015), and CYA is reduced to 4-cyadox monoxide and 1-cyadox monoxide and desoxycyadox. Moreover, the antibacterial activity of QdNOs is inhibited when the xanthine oxidase inhibitor, oxypurinol, is incubated with bacteria (Cheng et al., 2015). It is also found that cyadox can be enzymatically reduced to 4-cyadox monoxide and 1-cyadox monoxide by aldehyde oxidase and xanthine oxidase in the cytosol and by cytochrome b5 reductase in the microsomes of pig cells (Zheng et al., 2011). On the other hand, cyadox is only reduced to 4-cyadox monoxide in the non-enzymatic reduction mediated by heme groups of catalase and cytochrome P450 (Zheng et al., 2011). Our results suggest that CYA was reduced to 4-cyadox monoxide and desoxycyadox and OLA were reduced to 1-olaquindox monoxide and desoxyolaquindox in *C. perfringens* and *B. hyodysenteriae*. This may due to the enzymatic and non-enzymatic *N*-oxide reductive system in the two anaerobes that can reduce CYA and OLA in two different pathways.

In this study, both ROS and hydroxyl radicals were discovered in the two anaerobes treated with QdNOs (**Figure 1**). Early study has shown that radical scavengers can inhibit the death of *E. coli* cells and the generation of ROS (Cheng et al., 2015), suggesting that QdNOs kills bacteria by free radicals generated in bacteria during drug metabolism. Some other bactericidal antibiotics with diverse targets are thought to kill bacteria by inducing production of damaging reactive species (Kohanski et al., 2010; Dwyer et al., 2014). The ROS (mainly O_2_^-^, H_2_O_2_, and ⋅OH) in aerobes have been hypothesized to be originated from the interference of respiration by antibacterial agent action in the presence of oxygen (Dwyer et al., 2014). In *E. coli*, ⋅OH was not observed but a mass of O_2_^-^ (Cheng et al., 2015), this may be due to that OH was usually generated via the Fenton reaction which needs the participation of H_2_O_2_. H_2_O_2_ can be eliminated by catalase, thus the amount of ⋅OH was not obviously in *E. coli*.

Different from aerobic species, strictly anaerobic micro-organisms have no respiratory (electron-transport) chain (Thauer, 2015), so they cannot generate reactive products from reduction of O_2,_ such as H_2_O_2_. Therefore, the Fenton reaction would not occur without H_2_O_2,_ which affects the formation of hydroxyl free radical via Fenton reaction (Pesakhov et al., 2007). But according to early reports, it is possible that ⋅OH is a metabolic product of QdNOs under anaerobic conditions (Ganley et al., 2001), so in our study, ⋅OH was observed in the two anaerobes. This may be the reason why the two anaerobes are more sensitive to QdNOs than aerobic bacteria. Moreover, our results indicate that CYA and OLA generate ROS and ⋅OH much more efficiently than H_2_O_2_ whose concentration is much higher under the conditions tested. This is because that Fenton reaction is not effective enough in the two anaerobes. H_2_O_2_ and superoxide do not lead to oxidative damage to nucleotides, but ⋅OH is extremely toxic and will readily damage protein, membrane lipids, DNA and intracellular respiratory system, as well as cell wall.

Protection against free radicals in aerobes and facultative anaerobes is provided by some antioxidative enzymes, which are responsible for the elimination of ROS, the regulation of antioxidative defense and the synthesis of DNA related enzymes, such as DNA mismatch repair enzyme, *MutS* (Brioukhanov and Netrusov, 2004). Microorganisms with strong antioxidant defense system are thought to have a moderate or high tolerance to the oxidative environment compared to strictly anaerobes which lack an antioxidative enzymatic system or because of antioxidative enzymes displaying low activity (Benov and Fridovich, 1995). Our results suggest that QdNOs have a more effective antibacterial action against *B. hyodysenteriae* than *C. perfringens*, possibly because that *C. perfringens* is an aerotolerant anaerobe, which is more resistant to ROS than *B. hyodysenteriae*, a strict anaerobe.

The main role of cell wall is to maintain the cell shape to resist the intracellular osmotic pressure of bacteria (Cava et al., 2013), so the damage of bacterial cell wall leads to the change of cell shape (**Figure 4**). In this study, ALP assay results indicated that the cell wall damage was dose-dependent and compared with Gram-negative bacteria *B. hyodysenteriae* B204, Gram-positive bacteria *C. perfringens* CVCC1125 produced higher amounts of ALP (**Figure 2**). *Clostridium* organisms are endospore-forming bacteria and are able to differentiate into metabolically inert endospores typically, but not always, upon sensing unfavorable environmental conditions. Exposure to oxygen or other stresses may play a role in triggering the sporulation process (Al-Hinai et al., 2015). In our results, maybe it is oxidative stress caused by ROS that induced the sporulation process in *C. perfringens*. During sporulation process, bacteria may produce higher amounts of ALP (Chesnut et al., 1991) (**Figure 5**). Cell membrane also plays an important role in maintaining intracellular osmotic pressure. High level of ROS caused cell membrane damage and the leakage of the intracellular components (**Figures 3** and **4**).

The elongated and filamentous morphology of *E. coli* treated by quindoxin was considered to be caused by SOS response (Suter et al., 1978), which is an inducible DNA repair and damage tolerance system regulated mainly by *recA* and *lexA* (Craig and Roberts, 1980). *lexA* was up-regulated obviously in *E. coli* treated by CYA, and a number of SOS genes were induced in *E. coli* exposed to QdNOs (Cheng et al., 2015). The elongated and filamentous morphology of many bacteria cells was observed in the case of exposure to the DNA-damaging reagent. Several SOS response proteins are known to regulate cell division and induce filamentation in response to DNA damage and reactive oxidative intermediates, such as *YneA* in *Bacillus subtilis* and *SulA (SfiA)* in *E. coli* (Brune et al., 2005). Previous work in *E. coli* treated by CYA has shown that *sulA* expression is up-regulated in response to DNA damage (Cheng et al., 2015). In our results, both *C. perfringens* and *B. hyodysenteriae* treated by QdNOs were elongated and appeared filamentous morphology was seen (**Figure 4**; Supplementary Figure S5). This is consistent with previous research, indicating that QdNOs may also induce the SOS response in the two strains.

The presence of 8-OHdG in DNA indicates an oxidative stress which are resulting from ROS (Ihsan et al., 2011). CYA and OLA were less active against Gram-positive bacteria *C. perfringens* than Gram-negative bacteria *B. hyodysenteriae* (**Table 2**), and a higher 8-OHdG level was observed in *B. hyodysenteriae* than in *C. perfringens* (**Figure 6**).

Under anaerobic conditions, QdNOs are enzymatically reduced by bacterial metabolism to products that can interact with bacterial DNA, inducing mutations and DNA strand breaks (Sisson et al., 2000). In our study, QdNOs also induced chromosome DNA strand breaks and degradation (**Figure 7**). As reported for other bacterial species, an early event for VSH-1 induction by carbadox and H_2_O_2_ lead to a *recA*-centered SOS response (Auchtung et al., 2005). VSH-1 is induced to form viral particles after treatment with mitomycin C in *B. hyodysenteriae* and are released upon lysis of their host cell (Matson et al., 2005). In response to DNA damage caused by QdNOs, bacteria induce an SOS response to stimulate DNA repair. However, the SOS response may also induce prophage with production of infectious virions.

## Conclusion

Quinoxaline 1,4-di-*N*-oxides are a class of hypoxia-selective and redox-activated DNA damaging agents. Oxidative damage to DNA and damage of cell wall and cell membrane of *C. perfringens* and *B. hyodysenteriae* via ROS and hydroxyl radical generation occur during the metabolism process in bacteria under anaerobic conditions, resulting in the death of bacteria in the end. The level of intracellular ROS and 8-OHdG suggest that oxidative stress participates in antibacterial activity. Moreover, prophage VSH-1 was induced in *B. hyodysenteriae* cells. This study suggests that oxygen-free environment contributes to effective generation of free radicals during QdNOs metabolisms and the antimicrobial mechanisms of QdNOs against Gram-positive bacteria and Gram-negative bacteria are nearly the same.

## Author Contributions

FX contributed to design, data acquisition and analysis, data interpretation, drafted and critically revised the manuscript. GC contributed to the conception, design, data analysis, data interpretation, critically revised the manuscript and provided funding. YW, HH, XW, DC, DP, and ZL revised the manuscript. MD contributed to conception, and provided funding. ZY contributed to conception. All authors gave final approval to the manuscript and agree to be accountable for all aspects of the work.

## Conflict of Interest Statement

The authors declare that the research was conducted in the absence of any commercial or financial relationships that could be construed as a potential conflict of interest.

## Abbreviations

8-OHdG: 8-hydroxy-2′-deoxyguanosine
ALP: Alkaline phosphatase
ATCC: American Type Culture Collection
BHI: brain heart infusion
Cy1: desoxycyadox
Cy2: cyadox-1-monoxide
Cy10: cyadox- 4-monoxide
CYA: Cyadox
DCFH-DA: 2′,7′-dichlorofluorescin diacetate
HPF: 3′-(p-hydroxyphenyl) fluorescein
HPLC: High Performance Liquid Chromatography
IVDC: China Institute of Veterinary Drug Control
MIC: minimum inhibitory concentration
O1: olaquindox 4-monoxide
O2: desoxyolaquindox
O7: olaquindox-1-monoxide
OD: optical density
OLA: olaquindox
PBS: phosphate buffer saline
QdNOs: quinoxaline 1,4-di-*N*-oxide derivatives
ROS: reactive oxygen species
SD: Swine dysentery
