# Supplementary material for: Mechanisms of Antibacterial Action of Quinoxaline 1,4-di-N-oxides against Clostridium perfringens and Brachyspira hyodysenteriae
Source: Front Microbiol. 2016 Dec 5;7:1948. doi: 10.3389/fmicb.2016.01948 (PMC5147047; doi:10.3389/fmicb.2016.01948)
Supplement: Supplementary file 1 [file Data_Sheet_1.DOCX]

***Supporting data***


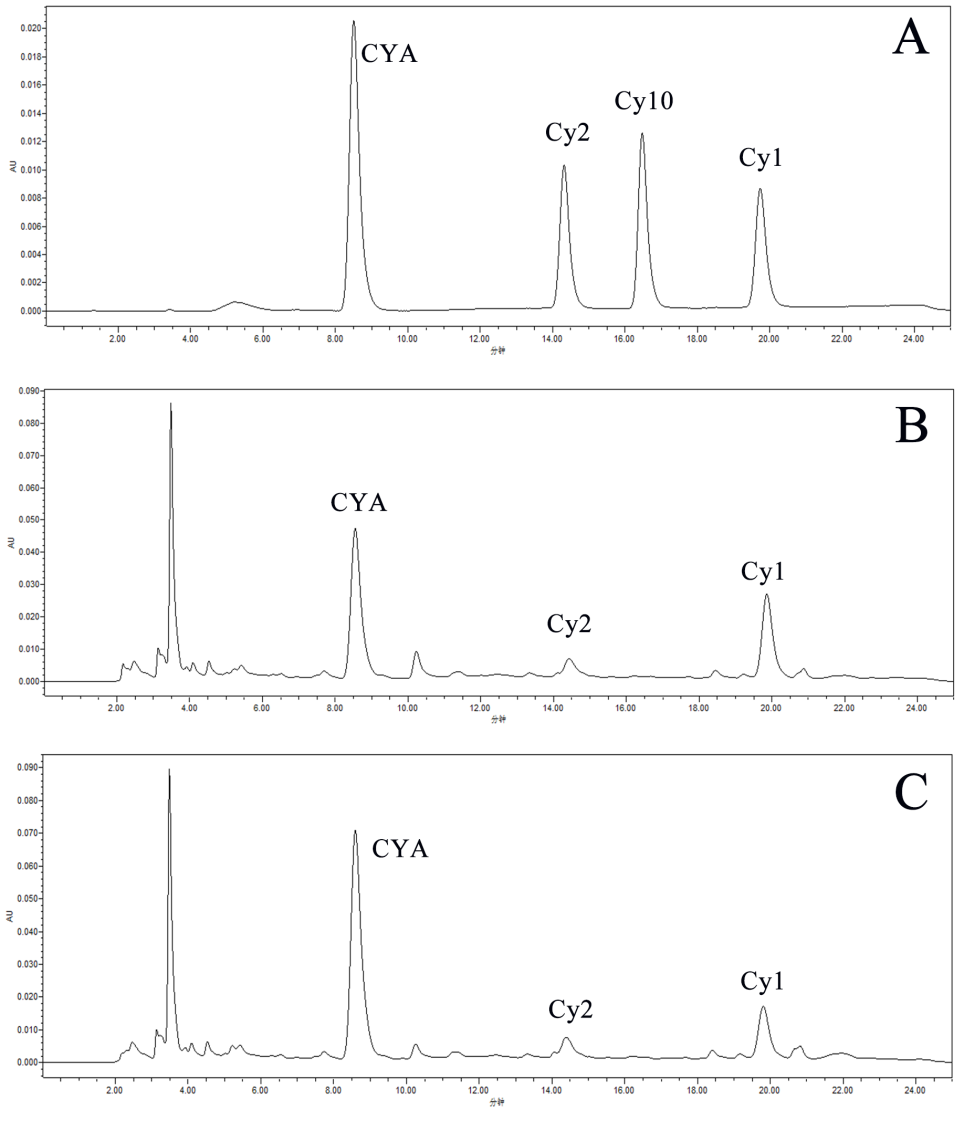


**Fig. S1 HPLC spectrum of CYA metabolites in *C. perfringens* CVCC 1125 (B) and *B. hyodysenteriae B204* (C) under anaerobic conditions**

(A) HPLC spectrum of standard substances of CYA and its metabolites. *C. perfringens* CVCC1125 cells were incubated with 4 μg/mL CYA (B) and *B. hyodysenteriae* B204 were incubated with 0.5 μg/mL CYA (C) under anaerobic conditions for 0.5 h. The extracts of the metabolites from the bacteria were subjected to HPLC as described in supplementary materials and methods.


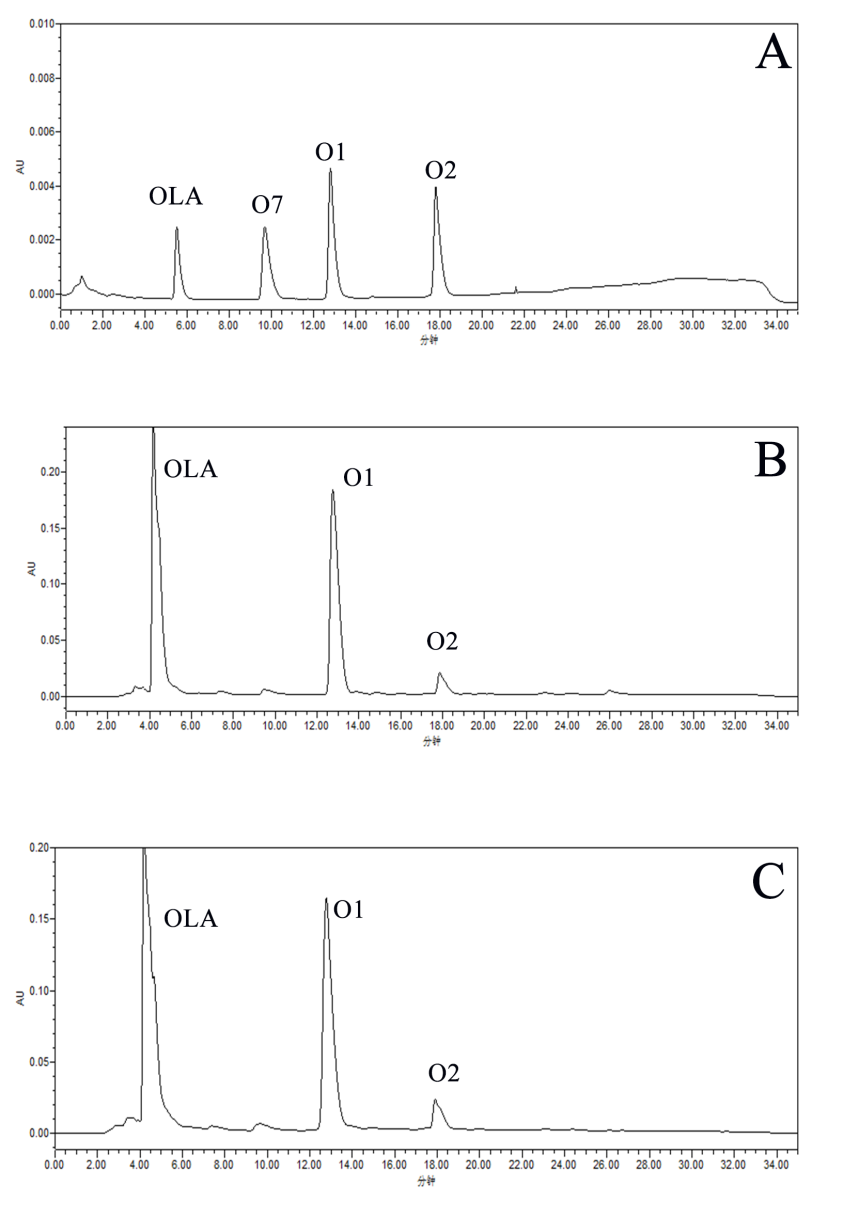


**Fig. S2 HPLC spectrum of OLA metabolites in *C. perfringens* CVCC 1125 (B) and *B. hyodysenteriae* B204 (C) under anaerobic conditions**

(A) Chromatograms of standard substances of OLA and its metabolites. *C. perfringens* CVCC1125 was incubated with 4 μg/mL OLA (B) and *B. hyodysenteriae* B204 was incubated with 1 μg/mL OLA (C) under anaerobic conditions for 0.5 h. The extracts of the metabolites from the bacteria were subjected to HPLC as described in supplementary materials and methods.

**
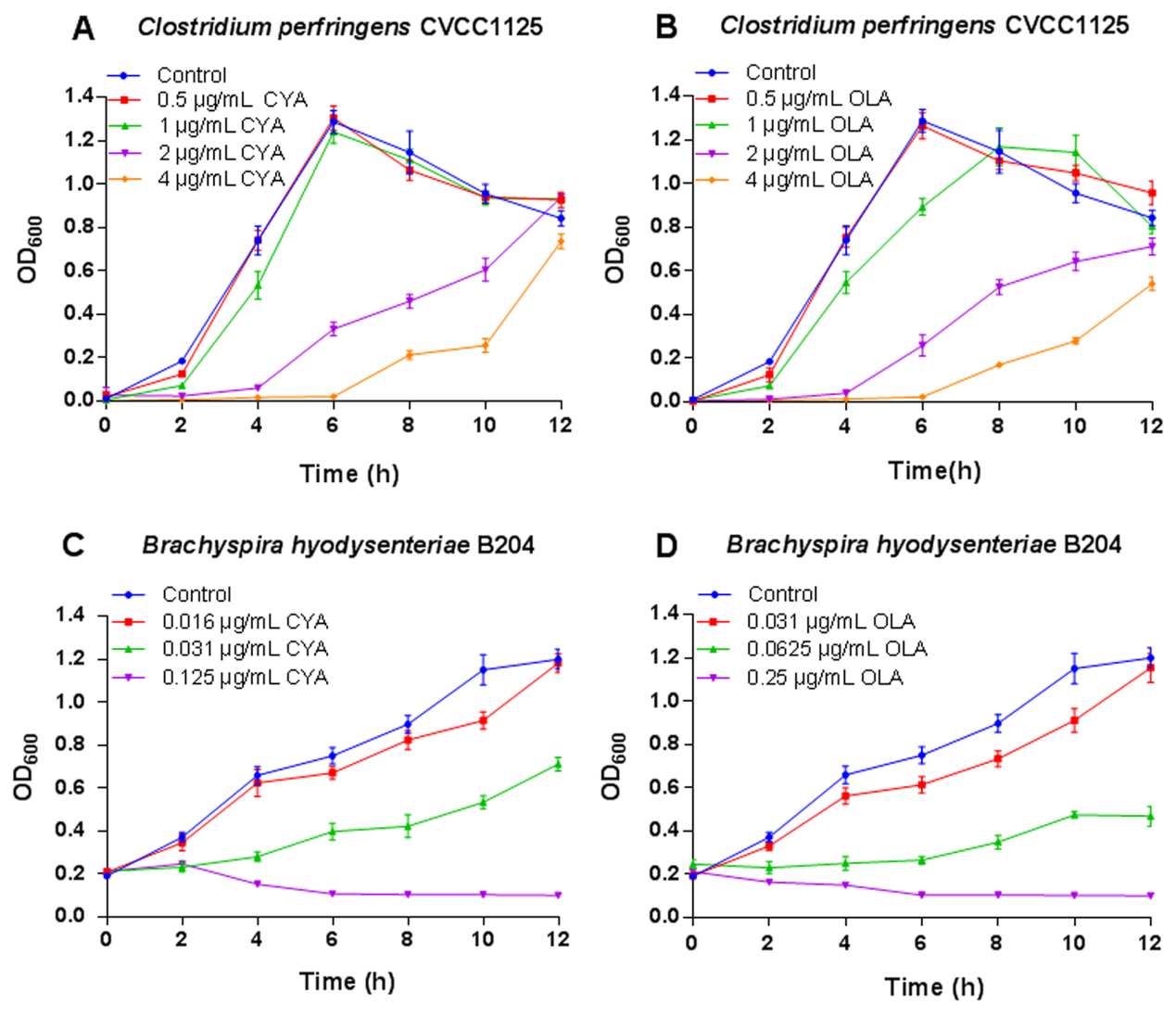
**

**Fig. S3 Effect of QdNOs on cell growth of *C. perfringens* CVCC 1125 (A and B) and *B. hyodysenteriae* B204 (C and D)**

(A) Under anaerobic conditions, *C. perfringens* CVCC1125 cells (2×10^5^ CUF/mL) were treated with indicated concentration of CYA. (B) Under anaerobic conditions, *C. perfringens* CVCC1125 cells (2×10^5^ CUF/mL) were treated with indicated concentration of OLA. (C) Under anaerobic conditions, *B. hyodysenteriae* B204 cells (10^7^ CUF/mL) were treated with indicated concentration of CYA. (D) Under anaerobic condition, *B. hyodysenteriae* B204 cells (10^7^ CUF/mL) were treated with indicated concentration of OLA. After incubation, OD_600_ of samples were detected every two hours as described in supplementary materials and methods.

**
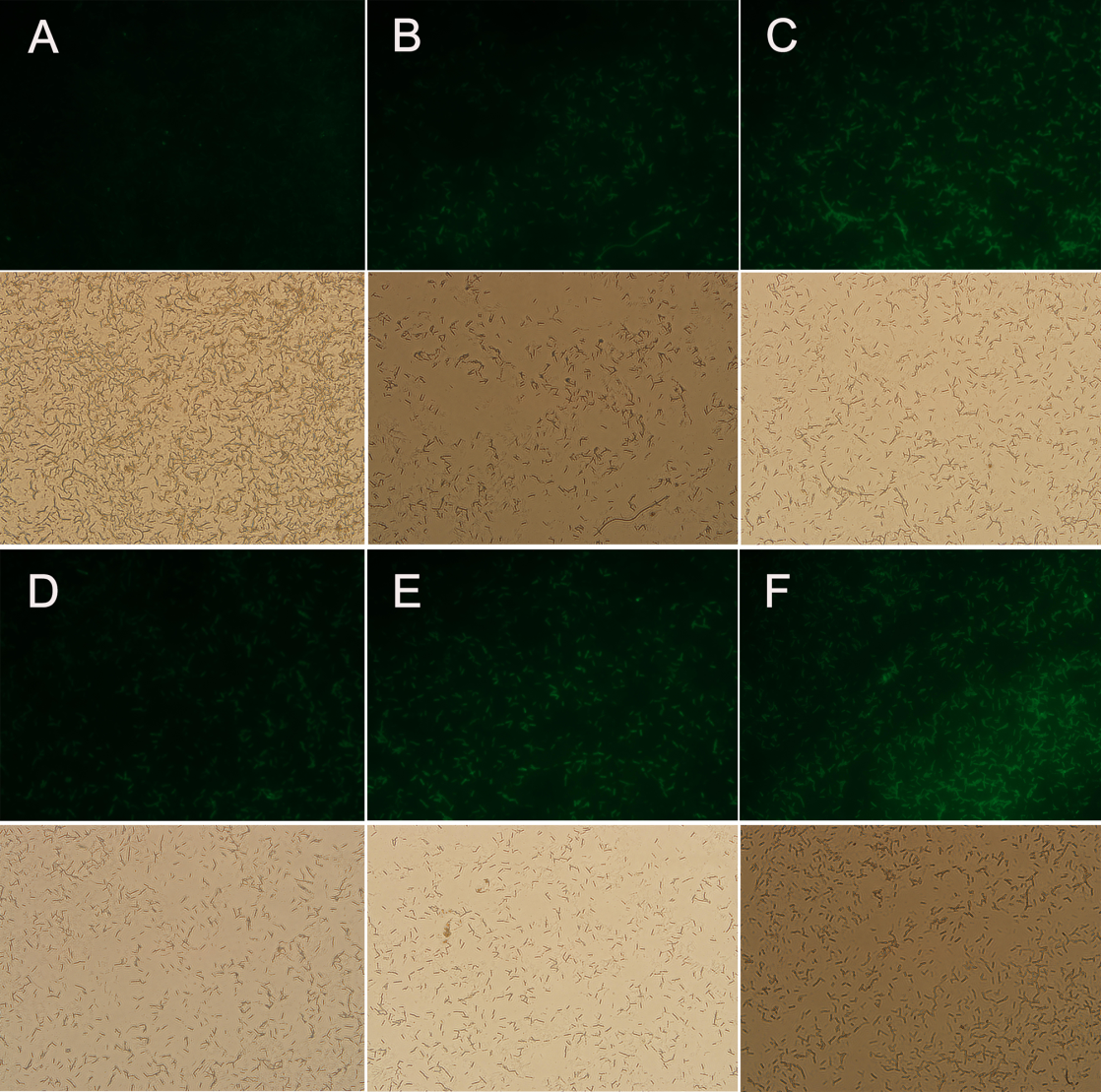
**

**Fig. S4 Hydroxyl radical in *C. perfringens* CVCC1125 exposed to QdNOs by fluorescence microscope under anaerobic conditions**

*C. perfringens* CVCC1125 cells were untreated (A) or treated with 1 µg/mL OLA (B), 4 µg/mL OLA (C), 1 µg/mL CYA (D), 4 µg/mL CYA (E), and 2.5 mM H_2_O_2_ as positive control (F) under anaerobic conditions for 1 h.


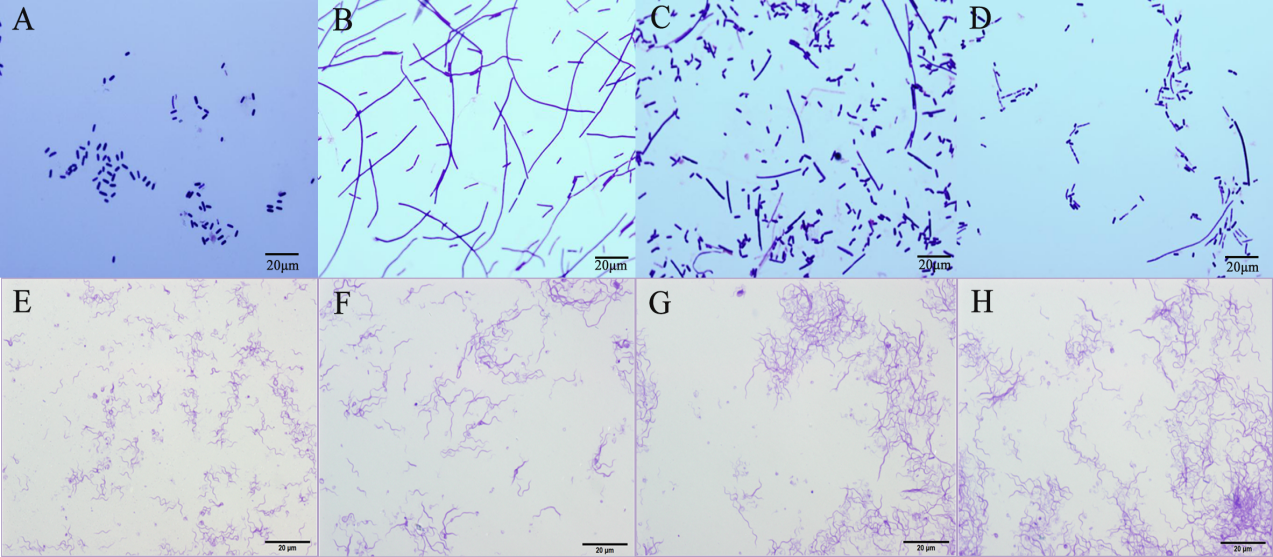


**Fig. S5 Morphology changes of *C. perfringens* CVCC1125 (A-D) and *B. hyodysenteriae* B204 (E-H) exposed to QdNOs by light microscope**

*C. perfringens* CVCC1125 cells were untreated (A) or treated with 0.25 µg/mL enrofloxacin (B), 0.5 µg/mL CYA (C), 0.5 µg/mL OLA (D) for 6 h under anaerobic conditions. *B. hyodysenteriae* B204 were untreated (E) or treated with 8 µg/mL enrofloxacin (F), 0.016 µg/mL CYA (G), 0.031 µg/mL OLA (H) for 24 h under anaerobic conditions.

***Supplementary materials and methods***

**Identification of CYA and OLA metabolites in *C. perfringens* and *B. hyodysenteriae***

*Clostridium perfringens* CVCC1125 and *B. hyodysenteriae* B204 were treated with 4 µg/mL (for *C. perfringens)* or 0.5 µg/mL (for *B. hyodysenteriae)* of CYA and 4 µg/mL (for *C. perfringens)* or 0.5 µg/mL (for *B. hyodysenteriae)* of OLA for 0.5 h under aerobic or anaerobic conditions (80% N_2_, 10% H_2_, and 10% CO_2_) respectively. 4 mL liquid supernatant of bacteria culture were added with 1 mL methanol and ultrasonicated for 2 min. After centrifugation, the supernatant was added with 1 ml of 15% trichloroacetic acid, followed by centrifugation at 10,000 g for 10 min. Then, the supernatant was dried under a stream of nitrogen, followed by addition of 500 μL initial mobile phase and mixed thoroughly. The solution was filtered through a 0.22 μm nylon membrane, and aliquots were subjected to the high performance liquid chromatography (HPLC) (Waters 2695, America) for analysis.

**Detection of effects of QdNOs on cell growth**

*C. perfringens* CVCC1125 was cultured to logarithmic phase, 15 mL fresh BHI was inoculated with 2% *C. perfringens* culture with different concentrations of drug, and incubated at 37˚C. The culture OD_600_ were monitored over time to detect differences in cell growth between treated and untreated group [1].

*B. hyodysenteriae* B204 cultures in early exponential growth phase (OD_600_ = 0.2) in BHIS broth were treated with different concentrations of drugs. OD_600_ the culture were monitored over time to detect differences in cell growth between treated and untreated cultures [2, 3].

**Cell morphology examination by light microscopy**

To examine phenotype changes in *C. perfringens* and *B. hyodysenteriae* cells induced by QdNOs at different concentrations, 10 mL samples were removed from the experimental and control wells at each time interval (6 h or 24 h incubation) from the test tube. The samples were examined on glass slides at 1000x magnifications under light microscope [4]. Images were viewed and photographed using the digital camera and built-in software.

**Detection of hydroxyl radical in *C. perfringens* by fluorescent microscope**

The bacterial culture was treated with QdNOs in anaerobic condition after incubation with drugs for the indicated times at 37°C, and then the reactions were stopped at 4°C. 3-(*p-*aminophenyl) fluorescein (APF) (Invitrogene, Canada) was used for the detection of hydroxyl radicals produced by QdNOs. A generation of hydroxyl radical was monitored by adding 10 μM of APF to each experimental test tube (containing bacterial inoculum and QdNOs dilutions) as well as the assay controls (bacterial inoculum). The negative control consisted of experimental test without APF. Then a spot of 100 μL bacteria sample was put on glass slide and the fluorescence was observed immediately under a fluorescent microscope (CX40, Olympus, Japan).

**Reference**

[1] Wang H, Zou D, Xie K, Xie M. Antibacterial mechanism of fraxetin against Staphylococcus aureus. Molecular medicine reports. 2014;10:2341-5.

[2] Stanton TB, Humphrey SB, Sharma VK, Zuerner RL. Collateral effects of antibiotics: carbadox and metronidazole induce VSH-1 and facilitate gene transfer among Brachyspira hyodysenteriae strains. Applied and environmental microbiology. 2008;74:2950-6.

[3] Matson EG, Thompson MG, Humphrey SB, Zuerner RL, Stanton TB. Identification of genes of VSH-1, a prophage-like gene transfer agent of Brachyspira hyodysenteriae. Journal of bacteriology. 2005;187:5885-92.

[4] Brudzynski K, Sjaarda C. Antibacterial compounds of Canadian honeys target bacterial cell wall inducing phenotype changes, growth inhibition and cell lysis that resemble action of beta-lactam antibiotics. PloS one. 2014;9:e106967.
